# MUC1 Contributes to BPDE-Induced Human Bronchial Epithelial Cell Transformation through Facilitating EGFR Activation

**DOI:** 10.1371/journal.pone.0033846

**Published:** 2012-03-22

**Authors:** Xiuling Xu, Lang Bai, Wenshu Chen, Mabel T. Padilla, Yushi Liu, Kwang Chul Kim, Steven A. Belinsky, Yong Lin

**Affiliations:** 1 Molecular Biology and Lung Cancer Program, Lovelace Respiratory Research Institute, Albuquerque, New Mexico, United States of America; 2 Department of Physiology and Lung Center, Temple University School of Medicine, Philadelphia, Pennsylvania, United States of America; Emory University, United States of America

## Abstract

Although it is well known that epidermal growth factor receptor (EGFR) is involved in lung cancer progression, whether EGFR contributes to lung epithelial cell transformation is less clear. Mucin 1 (MUC1 in human and Muc1 in animals), a glycoprotein component of airway mucus, is overexpressed in lung tumors; however, its role and underlying mechanisms in early stage lung carcinogenesis is still elusive. This study provides strong evidence demonstrating that EGFR and MUC1 are involved in bronchial epithelial cell transformation. Knockdown of MUC1 expression significantly reduced transformation of immortalized human bronchial epithelial cells induced by benzo[a]pyrene diol epoxide (BPDE), the active form of the cigarette smoke (CS) carcinogen benzo(a)pyrene (BaP)s. BPDE exposure robustly activated a pathway consisting of EGFR, Akt and ERK, and blocking this pathway significantly increased BPDE-induced cell death and inhibited cell transformation. Suppression of MUC1 expression resulted in EGFR destabilization and inhibition of the BPDE-induced activation of Akt and ERK and increase of cytotoxicity. These results strongly suggest an important role for EGFR in BPDE-induced transformation, and substantiate that MUC1 is involved in lung cancer development, at least partly through mediating carcinogen-induced activation of the EGFR-mediated cell survival pathway that facilitates cell transformation.

## Introduction

Lung cancer is a major health concern, afflicting approximately 160,000 people each year in the United States [1], [2]. Most lung cancers are associated with mainstream or sidestream cigarette smoke (CS). Carcinogens derived from CS such as benzo(a)pyrene (BaP) induce lung cancer through DNA damage. Because of activation of DNA repair pathways that remove genomic lesions and apoptosis that eliminates cells with extensive genetic damage, only a small fraction of cells acquiring DNA damage become malignant. Therefore, cancer development and progression likely depend on the balance between cell survival and apoptosis signals, both of which are activated by carcinogens and environmental factors. The pathways controlling survival include mitogen-activated protein kinases (MAPK), Akt and NF-κB [3], [4], [5]. Although we have learned a great deal about the tumor-promoting role of survival signaling, how CS activates these pathways in lung cancer initiation and progression remains poorly understood. Thus, delineating the mechanisms underlying the influences of survival signaling on cell transformation and tumor development could identify novel intervention targets for prevention and therapy for lung cancer.

Aberrant epidermal growth factor receptor (EGFR) activation is involved in cancer progression [6], [7], [8]. Lung cancer cells acquire dependence on EGFR activity for survival, substantiating the use of EGFR inhibitors for lung cancer therapy [9], [10]. The ligands for EGFR including EGF and transforming growth factor α (TGFα) bind EGFR, triggering EGFR dimerization and autophosphorylation. The autophosphorylated C-terminal tyrosine kinase domain of EGFR in the cytoplasm initiates a cascade of intracellular signaling pathways [12] [11], [12], [13]. The EGFR downstream signaling pathways include components of the Ras/Raf/MAPK (ERK, JNK and p38) and PI3K/Akt, of which ERK and Akt are two main kinases for EGFR-mediated cell survival and proliferation. The EGFR signaling is terminated by endocytosis of the phosphorylated receptor–ligand complex followed by proteasomal degradation of EGFR [12]. How EGFR is activated by carcinogen in lung epithelial cells and whether EGFR is required for lung epithelial transformation is not well understood. In a breast cancer mouse model, mucin 1 (MUC1 for human and Muc1 for nonhuman species) facilitated TGFα-induced EGFR activation and breast cancer development [14], [15]. Therefore, it is interesting to determine if MUC1 is also involved in carcinogen-induced EGFR activation for lung cancer development.

As a mucin family protein expressed on the bronchial epithelial cell membrane, MUC1 is induced during airway inflammation and plays an important role for the resolution of inflammation during respiratory tract infection [16], [17], [18], [19]. During chronic inflammation, MUC1 expression is sustained at a high level, which may contribute to cancer development [20]. MUC1 has two subunits that are coded by a single gene: the N-terminal subunit containing highly conserved repeats of 20 amino acids that are modified by O-glycosylation and the transmembrane C-terminal subunit containing 72 amino acids residues that binds to various proteins involved in signal transduction [20], [21]. MUC1 is regarded as a tumor antigen because it is aberrantly overexpressed in various cancers including lung cancer, and immunotherapy with anti-MUC1 antibodies showed substantial anticancer effect against prostate and breast cancers [16]. Although artificial overexpression of MUC1 triggers fibroblast cell transformation, the mechanism for this process is poorly understood [22]. While MUC1 interacts with a variety of cellular factors, recent studies have suggested functional interactions between MUC1 and EGFR-mediated survival signaling [14], [15], [20], [23], [24]. Interestingly, MUC1 expression levels were reported to be associated with response to EGFR inhibitors in lung cancer patients [25]. In non-small cell lung cancer, MUC1 is expressed in a depolarized pattern and correlated with poor patient survival [26]. Although it is known that MUC1 is involved in lung cancer progression particularly in metastasis and MUC1 is regarded as target for lung cancer therapy [16], [25], [27], [28], whether and how MUC1 contributes to CS-induced lung cancer initiation, particularly in lung epithelial cell transformation has not been well elucidated.

The goal of this study was to investigate the role and underlying mechanisms of MUC1 bronchial epithelial transformation. The results show that MUC1 contributes to the CS-specific carcinogen benzo(a)pyrene diol epoxide (BPDE)-induced human epithelial cell transformation through facilitating a cell survival pathway consisting of EGFR, Akt and ERK, highlighting that MUC1 and EGFR could be molecular targets for lung cancer prevention.

## Materials and Methods

### Reagents

BPDE was obtained from NCI Chemical Carcinogen Reference Standards Repository, Midwest Research Institute. Cycloheximide was purchased from Sigma. EGFR inhibitor II (a selective and irreversible inhibitor that blocks EGFR autophosphorylation), LY294002, U0126, and SP600125 were purchased from Calbiochem. Small interfering RNA (siRNA; SiGenome SMARTpool) for MUC1, EGFR and negative control siRNA were purchased from Dharmacon. The following primary antibodies were used for Western blot: anti-Mucin 1 (GP1.4, Santa Cruz), anti-β-Tubulin and -β-Actin (Sigma-Aldrich); and anti-phospho-EGFR (Y1086, Abcam), and anti-EGFR, -ERK, -Akt and -phospho-Akt (Ser473) (Cell Signaling Technology); anti-phospho-ERK (Y185/187) (Biosource). Filters collected from the AMESA Type 1300 smoking machine, which generated mainstream CS, were used to prepare cigarette smoke extract (CSE) by sequentially extracting materials from with culture medium (for dissolving water-soluble components) and DMSO (for dissolving water-insoluble components). The water-soluble and -insoluble fractions were mixed to make the total CSE before use. Total particulate material (TPM) was determined by weighing the filter before and after extraction. The MUC1 shRNA plasmid was constructed by inserting a synthetic oligonucleotide encoding a hairpin sequence with a 19-nucleotide stem that is homologous to the target sequence of human MUC1, CCGGGATACCTACCATCCTAT, and a 9-base loop sequence into pSilencer (Oligoengine, Seattle, WA).

### Cell Culture

BEAS-2B cells were obtained from American Type Culture Collection (ATCC) (Manassas, VA). The immortalized human bronchial epithelial cells, HBEC-2, were generously provided by Drs. Shay and Minna, Southwestern Medical Center, Dallas, TX [29]. BEAS-2B and HBEC cells were maintained in Keratinocyte serum free medium (KSFM) (Invitrogen), supplemented with 5 µg/L of human recombinant EGF and 50 mg/L of bovine pituitary extract in plates coated with fibronectin (Athena ES). The lung cancer cell lines (H23, A549, H460, H1299, H2009 and H1568) obtained from ATCC (Manassas, VA) were cultured in RPMI 1640 supplemented with 10% fetal bovine serum (FBS), 1 mM of glutamine, 100 U/ml of penicillin, and 100 µg/ml of streptomycin. Monolayer cultures were incubated at 37°C in a 95% humidified atmosphere containing 5% CO_2_.

### Soft agar assay

BEAS-2B cells were treated with BPDE (0.1 µM) every two days for a total of 3 treatments. For each treatment, the cells were exposed to BPDE for 1 h followed by incubation in fresh medium. The cells (1×10^4^/well, 12-well plates) were then seeded in soft agar for colony formation. Colonies in the agar were photographed and counted after 2 wk incubation. HBEC-2 cells were treated similarly as that of BEAS-2B cells with CSE (10 µg/ml TPM) or BPDE (0.1 µM) weekly (1 hr each time) for 12 wk; colonies were counted after 3 wk incubation [30]. The average number of colonies ± S.D. was determined using 6 randomly selected fields. All experiments were run in triplicate.

### Transfections

BEAS-2B cells were seeded in a 6-well plate at ∼50% confluency. After overnight culture, cells were transfected with small interfering RNA (siRNA) with INTERFERin™ siRNA transfection reagent (Polyplus-transfection) according to manufacturer's instructions. Forty-eight hours after transfection, MUC-1, EGFR, Akt and ERK protein levels were determined by Western blot. For stable shRNA transfection, cells were transfected with MUC1 shRNA at 30∼50% confluency using FuGENE HD transfection reagent (Roche) following the manufacturer's instructions. Clones stably expressing MUC1 knockdown were selected with puromycin (1 µg/ml) and identified by Western blot.

### Western Blot

Cells were harvested and total cell protein was extracted in M2 buffer (20 mM Tris-HCl, pH 7.6, 0.5% Nonidet P-40, 250 mM NaCl, 3 mM EDTA, 3 mM EGTA, 2 mM dithiothreitol, 0.5 mM phenylmethylsulfonyl fluoride, 20 mM-glycerophosphate, 1 mM sodium vanadate, and 1 µg/ml leupeptin). Equal amounts of cell proteins were resolved in 12% SDS-polyacrylamide gels and then transferred to PVDF membranes. The proteins were visualized by enhanced chemiluminescent detection reagent according to the manufacturer's instructions (Millipore).

### Cytotoxicity Assay

Cytotoxicity was assessed using a 3-(4,5-dimethylthiazolyl-2-)-2,5-diphenyltetrazolium bromide (MTT) cell proliferation assay and a lactate dehydrogenase (LDH) release-base cytotoxicity detection kit (Promega) [31]. Cells were seeded in 48-well plates at ∼70% confluency, cultured overnight, and then treated as indicated in the figure legends. Cell viability was determined using MTT assay. The percentage of viable cells was calculated using the following formula: Cell viability (%) = (Absorbance of treated sample/Absorbance of control) ×100. Cell death based on the release of LDH was calculated as follows: Cell death (%) = [(Experimental value-Spontaneous LDH release)/(Maximum LDH-Spontaneous LDH release)] ×100 [32].

### Statistics

All data were expressed as mean ± S.D. Statistical significance was examined by one-way analysis of variance. In all analyses, p<0.05 was considered statistically significant.

## Results

### MUC1 suppression inhibits BPDE-induced human bronchial cell transformation

**Figure 1:**
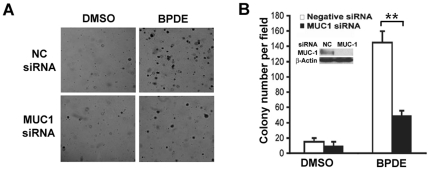
Suppression of MUC1 inhibits BPDE-induced transformation in BEAS-2B cells. *A*, BEAS-2B cells were transfected with MUC1 siRNA or negative control siRNA. The cells were then treated with BPDE (0.1 µM) for 1 wk and seeded in soft agar. Colony formation was photographed under a light microscope. *B*, Quantitative representation of the transformation experiment. Bars show the averages of colony numbers of 6 randomly selected fields. Data shown are mean ± S.D; ** P<0.01. Insert, Confirmation of MUC1 knockdown by Western blot.

Cell transformation, the transition of normal to cancerous cells, is a critical early process for cancer initiation. To investigate if MUC1 plays a role in bronchial epithelial cell transformation, MUC1 was knocked down by RNA interference in BEAS-2B, an immortalized human bronchial epithelial cell line that is sensitive to carcinogen-induced transformation *in vitro* [33]. The cells were treated with BPDE (0.1 µM) for 1 wk and then plated in soft agar for colony formation. Remarkably, transient knockdown of MUC1 expression strikingly reduced BPDE-induced colony formation. The negative control siRNA did not affect on BPDE-induced transformation (Figs. 1A, 1B). To exclude potential off-target effect of siRNA, this observation was validated by stably knocking down MUC1 expression in BEAS-2B cells with a MUC1 shRNA targeting different site in the MUC1 messenger RNA. A dramatic reduction of BPDE-induced colony formation was seen in cells transfected with MUC1 shRNA, but not the negative shRNA (Figs. S1). These results suggest that MUC1 plays a significant role in promoting BPDE-induced bronchial epithelial cell transformation.

### BPDE induces EGFR-mediated Akt and ERK activation

**Figure 2:**
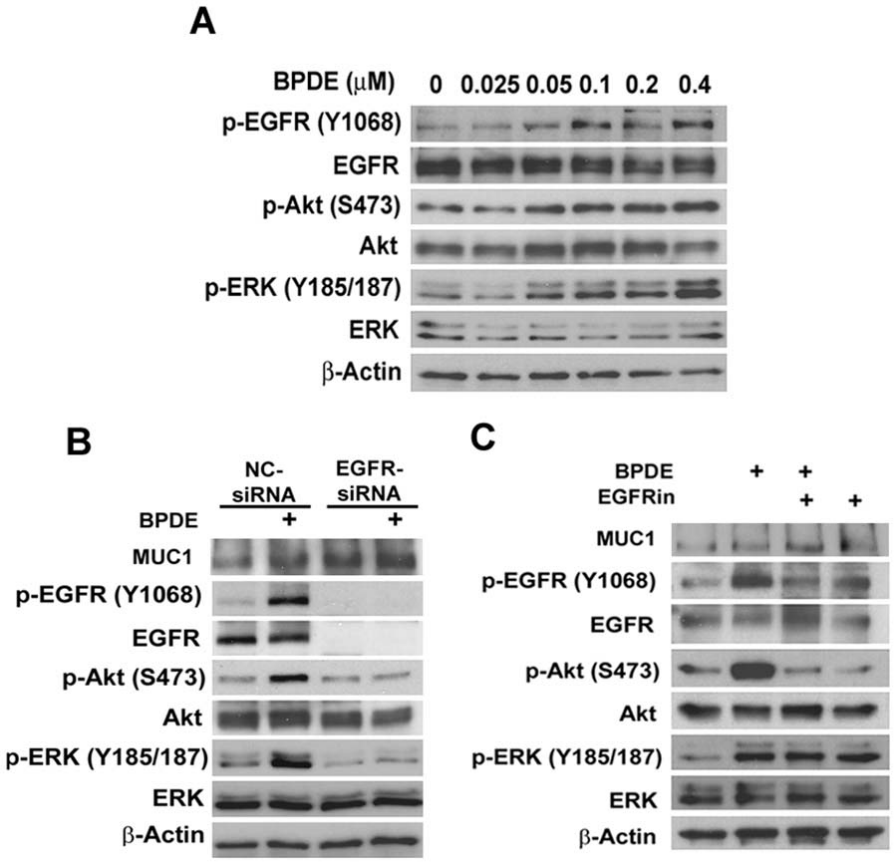
Transient BPDE exposure activates Akt and ERK through EGFR in human bronchial epithelial cells. *A*, Acute BPDE exposure induces activation of EGFR, Akt, and ERK in BEAS-2B cells. BEAS-2B cells were treated with the indicated concentrations of BPDE for 2 hr. Activation of EGFR, Akt and ERK were detected by Western blot with antibodies recognizing each phosphorylated protein (phosphorylation sites are indicated). β-Actin was detected as an input control. *B*, Suppression of EGFR attenuates the activation of Akt and ERK in BEAS-2B cells. The expression and activity of EGFR in BEAS-2B cells were suppressed with either EGFR inhibitor III or EGFR siRNA. The cells were then exposed to BPDE (0.4 µM) for 2 hr. The indicated proteins were detected by Western blot. β-Actin was detected as a loading control.

To investigate the underlying mechanism by which MUC1 potentiates BPDE-induced bronchial epithelial cell transformation, we focused on the activation of the EGFR pathway because this pathway can be activated by benzo(a)pyrene and MUC1 was reported to modulate EGFR activation in breast epithelial cells [34], [35]. HBEC-2 and BEAS-2B cells were treated with BPDE for 2 h and activation of EGFR was detected with an antibody recognizing EGFR phosphorylated at tyrosine 1068. BPDE activated EGFR in a dose-dependent manner, starting at a concentration of 0.05 µM (Fig. 2A). Akt and ERK, two major downstream kinases of EGFR, were activated by BPDE in a similar manner (Fig. 2A). Supporting this observation, cigarette smoke extract (CSE) activated EGFR, Akt and ERK in HBEC-2, starting 15 min and persisting over 2 h after treatment (Fig. S2A). Because Akt and ERK are activated by a variety of pathways other than EGFR, we examined if BPDE-induced Akt and ERK activation is mediated by EGFR by knocking down the expression of EGFR with EGFR siRNA. While the negative control siRNA had a marginal effect on BPDE-induced activation of Akt and ERK, the EGFR siRNA, which effectively eliminated EGFR expression, completely blocked BPDE-induced Akt and ERK activation (Fig. 2B). Consistently, the EGFR inhibitor, which effectively suppressed BPDE-induced EGFR activation, effectively attenuated BPDE-induced Akt and ERK activation (Fig. 2C). Neither EGFR siRNA nor the EGFR inhibitor inhibited the expression of MUC1 (Figs. 2B, 2C), suggesting that it is unlikely that MUC1 functions downstream of EGFR for BPDE-induced Akt and ERK activation. Altogether, these results strongly suggest that BPDE activates EGFR, which in turn, mediates activation of Akt and ERK pathways in bronchial epithelial cells.

**Figure 3:**
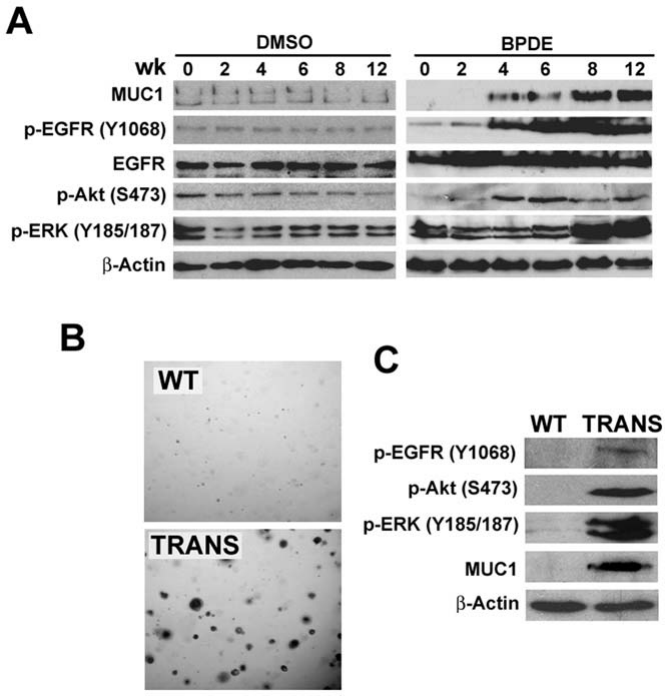
Chronic BPDE exposure activates Akt and ERK through EGFR in human bronchial epithelial cells. *A*, Induction of MUC1 expression and EGFR-, Akt- and ERK-activation in HBEC-2 cells by BPDE. Specifically, HBEC-2 cells were treated with the vehicle DMSO or BPDE (0.1 µM) for the indicated weeks. Western blot was the same as in *A*. β-Actin was detected as a loading control. *B*, Increased MUC1 expression and EGFR-, Akt- and ERK-activation in transformed HBEC-2 cells by BPDE. HBEC-2 cells were treated with BPDE (0.1 µM) for 12 wk and then seeded in soft agar. Colonies were grown up for 3 wk in transformed cells (TRANS). Wild-type (WT, exposed to sham) HBEC-2 cells were as a negative control. Expression of MUC1 and activation of Akt and ERK were detected by Western blot in both WT and the transfected cells. β-Actin was detected as a loading control.

Because transformation of HBEC-2 is a slow process that takes up to 12 wk [30], [36], we then examined if chronic exposure to a non-cytotoxic concentration of BPDE that induces transformation results in EGFR activation. Cells were treated weekly with BPDE (0.1 µM) for up to 12 wk and activation of EGFR, Akt and ERK was detected by Western blot. Activation of EGFR was first detected 4 wk post treatment, gradually increased and peaked after 8 wk and was sustained at 12 wk (Fig. 3A). A similar trend of activation of Akt and ERK was also observed. EGFR, Akt and ERK activation was detected in the cells after several passages, suggesting a sustained constitutive activation of EGFR-mediated pathways. Interestingly, the expression level of MUC1 is also induced at 4 wk and gradually increased through 12 wk (Fig. 3A). Consistent with the results from BPDE treatment, chronic CSE exposure similarly activated EGFR, Akt and ERK activation, which was also associated with increased MUC1 expression (Fig. S2B). These results suggest that chronic exposure to BPDE results in constitutive activation of EGFR, Akt, and ERK, which is associated with increased expression of MUC1 in HBEC-2 cells. To further substantiate the finding that EGFR is activated during transformation, we examined this pathway in precancerous cells HBEC-2B, which were derived from colonies that formed in soft agar after chronic BPDE exposure [30], [36]. Although these cells were unable to form tumors in nude mice [30], [36], they readily form colonies in soft agar (Fig. 3B). In these cells, EGFR, Akt and ERK were constitutively activated in association with MUC1 overexpression (Fig. 3C). The association of MUC1 expression and EGFR activation suggests that MUC1 plays a role in BPDE-induced EGFR activation and cell transformation.

### EGFR-mediated Akt and ERK activation protects cells against BPDE-induced cytotoxicity

**Figure 4:**
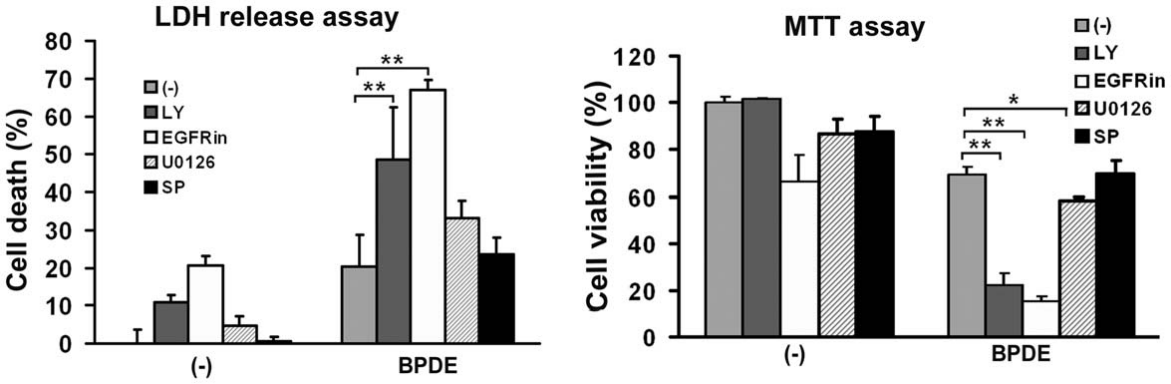
Blocking EGFR, Akt and ERK activation potentiates BPDE-induced cytotoxicity. BEAS-2B cells were pretreated with the indicated inhibitors (LY (10 µM) for Akt, EGFRin (6 µM) for EGFR, U0126 (5 µM) for ERK, and SP (10 µM) for JNK) for 30 min followed by exposure to BPDE (0.2 µM) for 48 hr. Cell viability was detected by LDH release and MTT assays. Data shown are mean ± S.D; ** P<0.01, * P<0.05.

Because survival of cells with genetic mutations is essential for cancer initiation and Akt and ERK are well known survival signals, we next investigated if BPDE-induced and EGFR-mediated Akt and ERK activation protects cells from BPDE-induced cytotoxicity. Pharmacological inhibitors blocking each pathway were followed by treatment with BPDE at a concentration (0.2 µM) that induced moderate cell death. Under this condition, BPDE caused ∼20% cell death that was detected by LDH release and MTT assays. While the EGFR inhibitor alone was slightly toxic, it dramatically increased cell death. Coordinately, the PI3 kinase (PI3K)/Akt inhibitor LY294002 effectively potentiated BPDE-induced cytotoxicity. Surprisingly, the MEK/ERK inhibitor U0126 had only a slight sensitization to BPDE-induced cell death. As a control, the JNK inhibitor SP600125 had little effect on BPDE's cytotoxicity (Fig. 4A, 4B). All the inhibitors were effective in blocking their respective pathways (data not shown) [37]. These results suggest EGFR-mediated pathways, particularly Akt, play important roles in protecting bronchial epithelial cells against BPDE-induced cell death, which may contribute to lung cancer development.

### Suppression of EGFR, Akt and ERK activation inhibits BPDE-induced cell transformation

**Figure 5:**
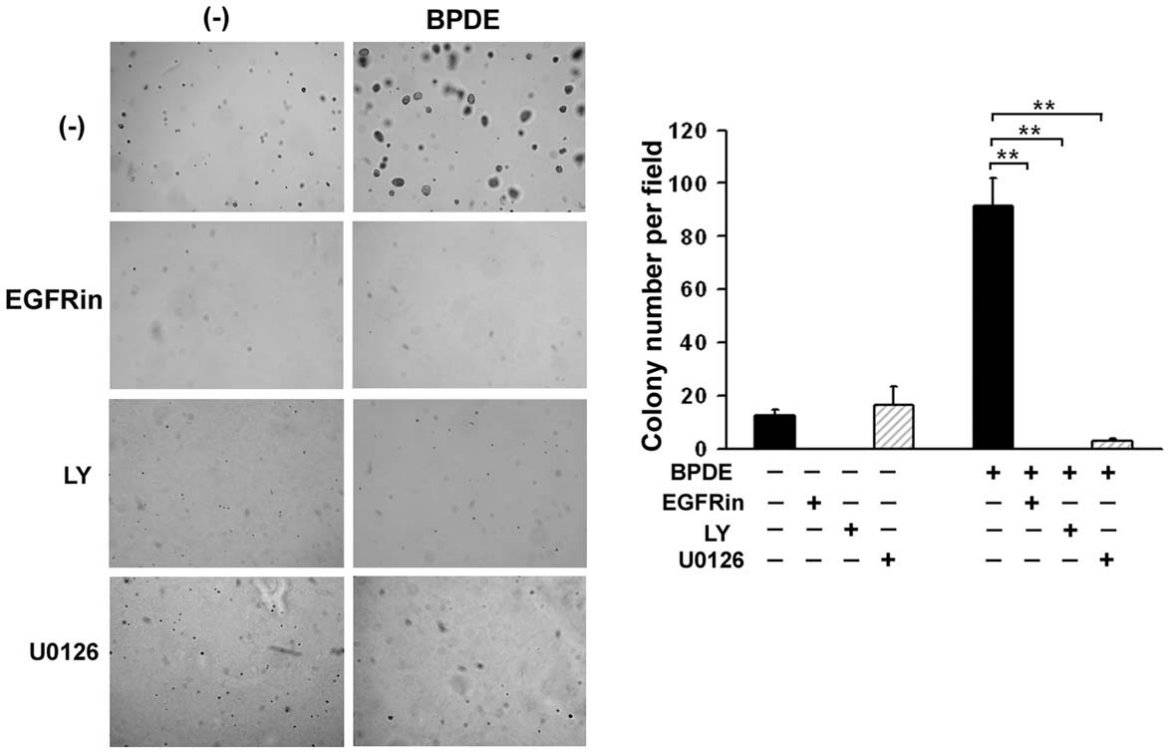
Suppression of EGFR, Akt and ERK activation inhibits BPDE-induced cell transformation. Graphical and quantitative representation of colony formation in soft agar of BEAS-2B cells exposed to BPDE (0.1 µM) and/or the indicated inhibitors (EGFRin, LY, and U0126) every two days for 1 week. Bars show the averages of colony numbers of 6 randomly selected fields. Data shown are mean ± S.D; ** P<0.01.

Next, we assessed the role of EGFR, Akt and ERK in BPDE-induced cellular transformation. BEAS-2B cells were treated with BPDE (0.1 µM), with or without inhibitors against each pathway for 1 wk, and then plated in soft agar for colonies formation. As expected, inhibition of EGFR and Akt effectively suppressed BPDE-induced colonies formation. Surprisingly, the ERK inhibitor U0126, which only slightly increased BPDE's cytotoxicity, effectively blocked BPDE-induced transformation (Fig. 5A, 5B). This observation suggests that ERK contributes to cell transformation by a mechanism other than cell survival. Nevertheless, these results suggest that activation of EGFR, Akt and ERK is required for transforming human bronchial epithelial cells.

### MUC1 is required for EGFR stabilization and BPDE-induced EGFR, Akt and ERK activation

**Figure 6:**
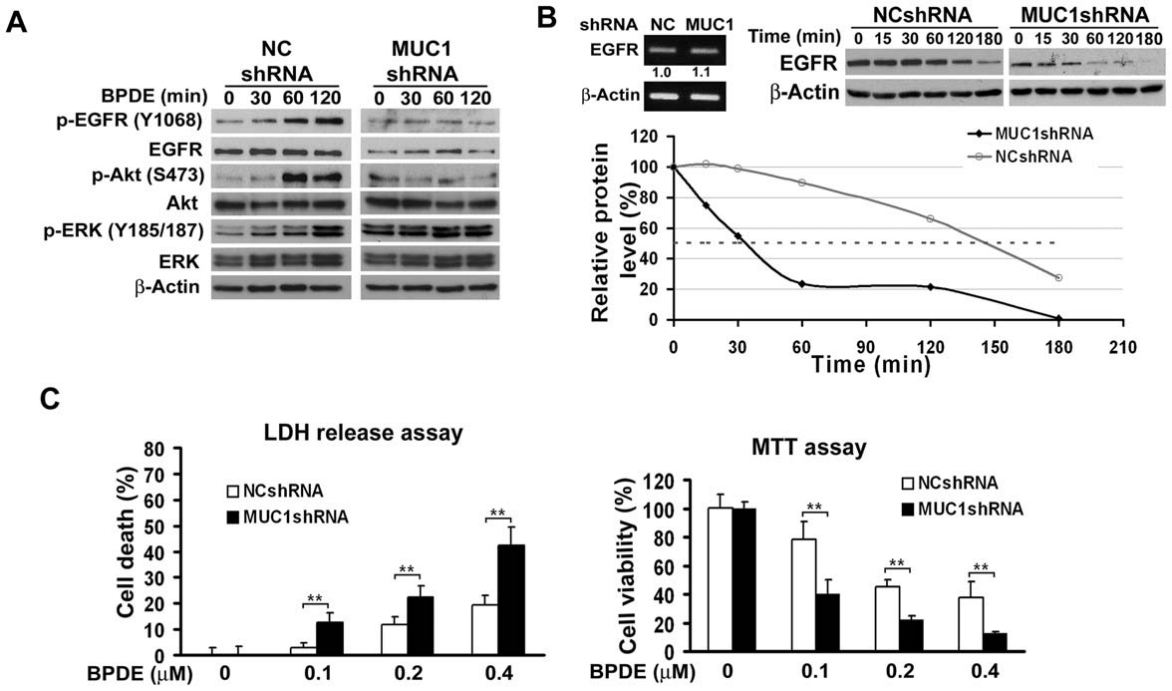
MUC1 stabilizes EGFR, contributes to BPDE-induced EGFR, Akt and ERK activation in BEAS-2B cells and protects cells from BPDE-induced cytotoxicity. *A*, MUC1 is required for BPDE-induced EGFR, Akt and ERK activation. Cells stably transfected with MUC1 shRNA or negative control shRNA were treated with BPDE for the indicated time periods. Activation of each protein was detected with antibodies against the phosphorylated form of the proteins. The phosphorylation sites of each protein are indicated. Total EGFR, Akt and ERK were also detected. β-Actin was detected as an input control. *B*, Reduced EGFR expression in MUC1 Knockdown cells. *Upper left*, equal amounts of total RNA from the indicated cells were detected for EGFR mRNA expression. β-Actin was detected as an input control. *Upper right*, Cells stably transfected with MUC1 shRNA or negative control shRNA were treated with cycloheximide (CHX, 10 µM) for the indicated time periods. EGFR protein was detected by Western blot. β-Actin was detected as an input control. *Lower right*, quantification of the results of *Upper right*. The intensity of the individual bands was quantified by densitometry (NIH Image 1.62) and normalized to the corresponding input control (β-actin) bands. EGFR expression changes were calculated with the control taken as 100%. *C*, BPDE-induced cytotoxicity is increased in MUC1 knockdown cells. BEAS-2B WT and MUC1 knockdown cells were treated with the indicated concentrations of BPDE for 48 hr. Cell viability was detected by LDH release and MTT assays. Data shown are mean ± S.D; **P<0.01.

Because BPDE stimulated EGFR-mediated Akt and ERK activation that was associated with MUC1 overexpression (Figs. 3A, 3C) and MUC1 modulates EGFR activation in breast epithelial cells [34], [35], we examined if MUC1 is involved in BPDE-induced activation of EGFR-mediated Akt and ERK pathways. BEAS-2B cells with stable MUC1 knockdown were compared to the cells stably transfected with a negative shRNA vector with respect to BPDE-induced EGFR, Akt and ERK activation. Although the phosphorylation of EGFR, Akt and ERK was substantially activated by BPDE in the control cells, MUC1 knockdown completely attenuated BPDE-induced activation of these pathways (Fig. 6A). These results strongly suggest that MUC1 is required for BPDE-induced activation of EGFR, Akt and ERK in bronchial epithelial cells. It is worthy noting that the basal pERK was remarkably higher in the MUC1 knockdown cells and the extent of suppression of BPDE-induced ERK activation was lower than that of Akt. This may be explained by the complexity of ERK activation in cells and other pathway(s) may compensate the suppression of EGFR-mediated ERK activation.

It was noticed that the expression of EGFR protein was reduced in the MUC1 knockdown cells (Fig. 6A). However, the EGFR message RNA expression levels were comparable between the cells with or without MUC1 knockdown (Fig. 6B). Therefore, we examined if the stability of EGFR is regulated by MUC1. Protein synthesis was shut off with cycloheximide and MUC1 expression was monitored for up to 3 hr. The half-life of EGFR in the MUC1-suppressed cells was ∼32 min, much shorter than that of the negative control shRNA transfected cells (∼145 min) (Fig. 6B). The mechanism of EGFR degradation is complex, which involves a pathway involving both lysosome and proteasome [38]. Consistently, the proteasome inhibitor MG132 and lysosome inhibitor chloroquine alone or in combination eliminated the EGFR expression difference between the MUC1 knockdown and control cells (Fig. S3). These results imply that decreased EGFR protein stability contributes to decreases of this protein expression in MUC1-suppressed cells, which leads to the suppression of BPDE-induced activation of EGFR, Akt and ERK.

### Suppressing MUC1 potentiates BPDE-induced cytotoxicity

Having established that MUC1 is required for BPDE-induced activation of EGFR-mediated Akt and ERK that protects bronchial epithelial cells against BPDE's cytotoxicity, we further investigated if MUC1 plays a survival role when cells are exposed to BPDE. BEAS-2B cells with stable MUC1 knockdown and control cells transfected with negative shRNA were treated with different concentrations of BPDE and cytotoxicity was detected by LDH release and MTT assays. The results clearly show that knockdown of MUC1 substantially potentiated BPDE-induced cell death (Fig. 6C). Because in the MUC1 knockdown cells BPDE-induced activation of EGFR-mediated Akt and ERK is suppressed (Fig. 6A) and these pathways are required for cell survival during BPDE challenge and BPDE induced transformation (Figs. 4, 5), it is likely that MUC1 protects the cells from BPDE-induced cytotoxicity and promotes cell transformation through potentiating BPDE-induced activation of EGFR-mediated survival pathways.

## Discussion

**Figure 7:**
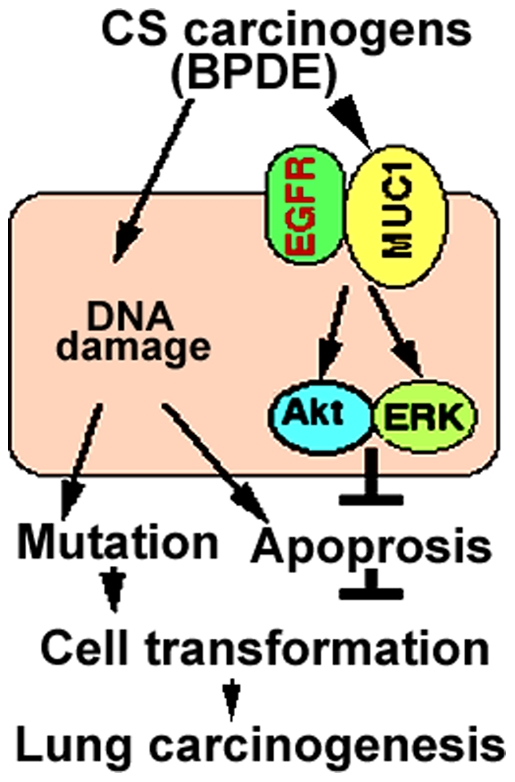
A model of MUC1-mediated EGFR activation and HBEC transformation. CS carcinogens such as BPDE trigger MUC1 expression in bronchial epithelial cells, facilitating EGFR-mediated cell survival signaling via Akt and ERK activation. Akt and ERK protect cells against DNA damage-mediated apoptosis to promote cell transformation, facilitating lung carcinogenesis.

This study provides strong evidence supporting a cancer-promoting role of MUC1 in CS-induced lung carcinogenesis: MUC1 potentiated transformation of human bronchial cells induced by the tobacco carcinogen BPDE; BPDE markedly activated EGFR and its downstream pathways Akt and ERK, and blocking these pathways significantly increased BPDE-induced cytotoxicity and inhibited cell transformation; Suppression of MUC1 expression destabilized EGFR protein and inhibited BPDE-induced activation of EGFR, Akt and ERK, and subsequently increased BPDE-induced cell death. These results suggest that BPDE-induced transformation requires EGFR-mediated Akt and ERK activation, and that MUC1 plays a lung cancer-promoting role during lung cancer development, at least partly through mediating carcinogen-induced activation of the EGFR-mediated cell survival pathways that neutralize carcinogen's cytotoxicity to facilitate cell transformation (Fig. 7).

Although the role of EGFR in lung cancer progression is well known, whether and how EGFR contributes to lung epithelial cell transformation is less clear. Specifically, whether EGFR is involved in CS-induced transformation of lung epithelial cells has not been reported. We found that CSE or the tobacco specific carcinogen BPDE activate EGFR, and blocking EGFR signaling effectively suppressed BPDE-induced transformation. These results clearly place EGFR as a pivotal factor for lung cancer development at the early time point. Our results further demonstrate that the EGFR-mediated signaling pathways, particularly Akt, prevent cells from BPDE's cytotoxic effect and thereby potentiate bronchial epithelial cell transformation. The ERK pathway, which contributed moderately to cell survival, significantly potentiated cell transformation presumably through a mechanism other than cell survival. In addition to cell survival, ERK is a major pathway for cell proliferation [39]. Indeed, proliferation of mutated cells after acquiring genetic and epigenetic modifications for malignant transformation is vital for giving rise to tumors. Therefore, it remains to be determined if ERK potentiates cell transformation through stimulating transformed cell proliferation. Nevertheless, our results suggest that activation of EGFR is a requisite for tobacco carcinogens such as BPDE to induce malignant transformation.

Although it is known that CS activates EGFR in lung epithelial cells, the underlying mechanism is not well elucidated. It has been shown that hydrogen peroxide-mediated stabilization of EGFR is involved [40]. Here we show evidence that EGFR is activated by CS specific carcinogen BPDE and MUC1 is required for the activation. CS-induced MUC1 distribution is related to EGFR activation in human bronchial epithelial cells [41]. Because EGFR mRNA level was not affected by the expression of MUC1, it is unlikely that MUC1 impacts EGFR at the level of transcription. Instead, posttranslational modulation of EGFR is likely involved. Indeed, reduced EGFR protein expression was observed in the MUC1 knockdown cells. We clearly show that the stability of EGFR protein is reduced in MUC1 knockdown cells, suggesting that MUC1 potentiates BPDE-induced EGFR activation through stabilization of the latter, similarly as in ligand-induced EGFR activation in breast cancer cells [23]. Furthermore, although we have not detected obvious induction of EGF or TGFα autocrine from bronchial epithelial cells by BPDE (data not shown), whether other EGFR ligands are involved in BPDE-induced and MUC1-potentiated EGFR activation deserves further study.

It is well known that MUC1 expression in bronchial epithelial cells is induced during inflammation [17], and chronic inflammation is associated with CS-induced lung carcinogenesis [42], [43]. Therefore, it is plausible to hypothesize that persistent MUC1 expression during CS-induced chronic inflammation promotes lung carcinogenesis. As MUC1 potentiates BPDE-induced activation of EGFR, it is likely that MUC1 contributes to lung cancer development at least in part through EGFR-mediated cell survival pathways such as Akt and ERK. While TNFα plays an important role in inducing MUC1 expression in human lung epithelial cells during acute inflammation as well as in lung infection [44], [45], how chronic inflammation enhances MUC1 expression has not been determined. It is possible that consistent stimulation by TNFα secreted from inflammatory cells keeps MUC1 expression in bronchial and alveolar cells at high levels to promote lung cancer development [46], [47]. However, other mechanisms might underlie. Indeed, in our *in vitro* assays with HBEC alone, MUC1 expression was induced by BPDE or CSE. MUC1 retained at a high level when cells were transformed, consistent with that MUC1 is constitutively overexpressed in lung cancers. Thus, the mechanisms by which MUC1 expression is activated by carcinogens and retained at high levels in transformed cells deserve further investigation. Nevertheless, our results suggest that MUC1 functions as a mediator that bridges CS-induced pulmonary inflammation and lung cancer development by potentiating EGFR-mediated survival signaling.

Eliminating transformed cells at the early stage of tumor initiation would be a good chemoprevention approach against cancer. In this regard, MUC1 and EGFR may serve as molecular targets for lung cancer prevention. It has been shown that targeting EGFR is able to reduce lung cancer burden in animal studies [48]. Although targeting MUC1 and EGFR individually have been proven to be effective in treating a portion of lung cancer patients, chemoprevention against these two factors has not been studied. In addition, because combined chemoprevention targeting more than one oncogenic protein could potentiate the cancer preventive activity and reduce toxicity, it would be interesting to determine if a regime targeting both MUC1 and EGFR achieves more effective prevention against lung cancer.

## Supporting Information

Figure S1 **Stable knockdown of MUC1 inhibits BPDE-induced transformation in BEAS-2B cells.** BEAS-2B cells were infected with MUC1 shRNA or negative control shRNA and stable clones were selected. Cell transformation and data analysis are the same as described in Fig. 1 A and B. Insert, Confirmation of MUC1 knockdown by Western blot.
(TIF)

Figure S2 **Induction of MUC1 expression and EGFR-, Akt- and ERK-activation in HBEC-2 cells by CSE exposure.** **A,** HBEC-2 cells were treated with CSE (10 µg/ml TPM) for the indicated time periods. Activation of each protein was detected with antibodies against the phosphorylated form of the proteins. The phosphorylation sites of each protein were indicated. Total EGFR was also detected. β-Actin was detected as a loading control. **B,** HBEC-2 cells were treated with CSE (10 µg/ml TPM) for the indicated weeks. Western blot was same as in A. The expression of MUC1was also detected. β-Actin was detected as a loading control.
(TIF)

Figure S3 **Blocking protein degradation with MG132 and chloroquine eliminates EGFR expression difference between the MUC1 knockdown and control cells.** Beas-2B cells stably transfected with MUC1 shRNA or negative control (NC) shRNA were treated with lysosome inhibitor chloroquine (CQ, 20 µM), proteasome inhibitor MG132 (10 µM), or both for 15 h. EGFR protein was detected by Western blot. β-Actin was detected as an input control.
(TIF)
