# Supplementary figures and images for: MUC1 Contributes to BPDE-Induced Human Bronchial Epithelial Cell Transformation through Facilitating EGFR Activation
Source: PLoS One. 2012 Mar 22;7(3):e33846. doi: 10.1371/journal.pone.0033846 (PMC3310874; doi:10.1371/journal.pone.0033846)

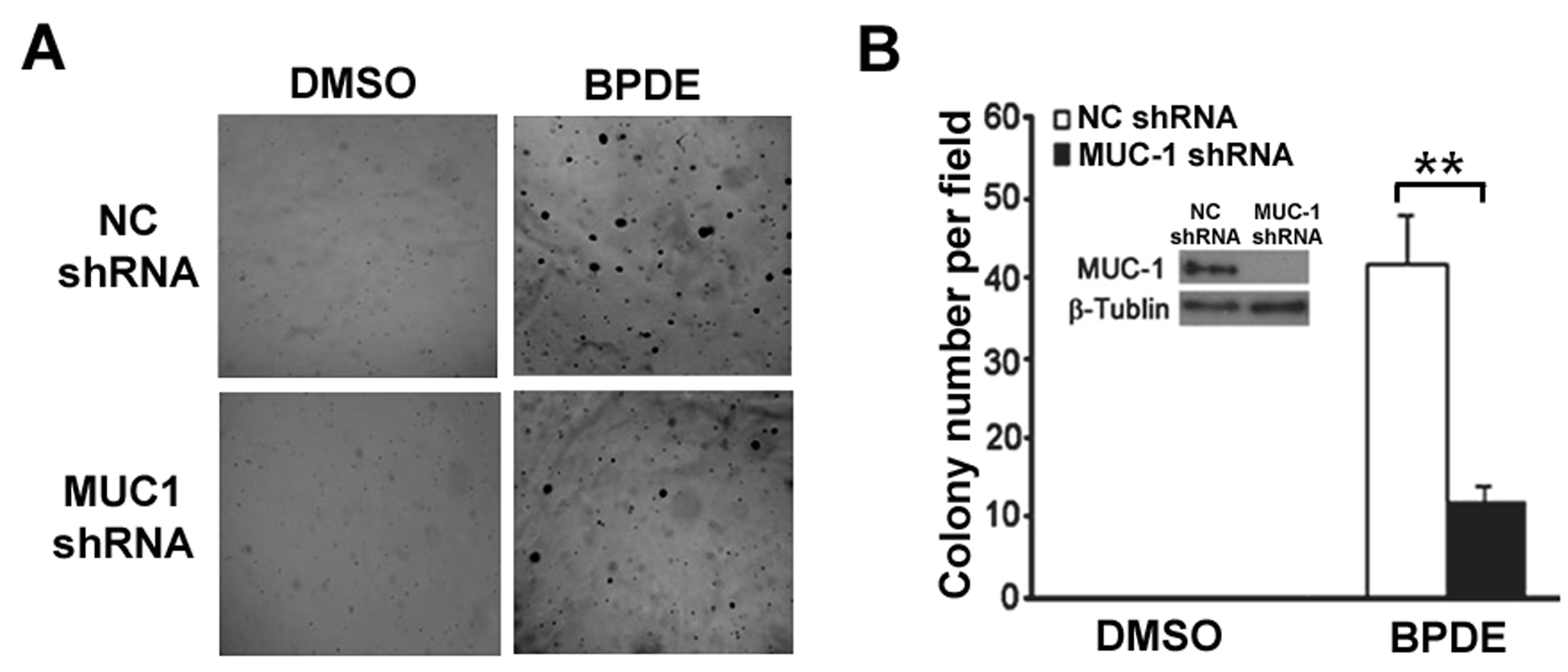

Supplement: Figure S1 — Stable knockdown of MUC1 inhibits BPDE-induced transformation in BEAS-2B cells. BEAS-2B cells were infected with MUC1 shRNA or negative control shRNA and stable clones were selected. Cell transformation and data analysis are the same as described in Fig. 1 A and B. Insert, Confirmation of MUC1 knockdown by Western blot. (TIF) [file pone.0033846.s001.tif]

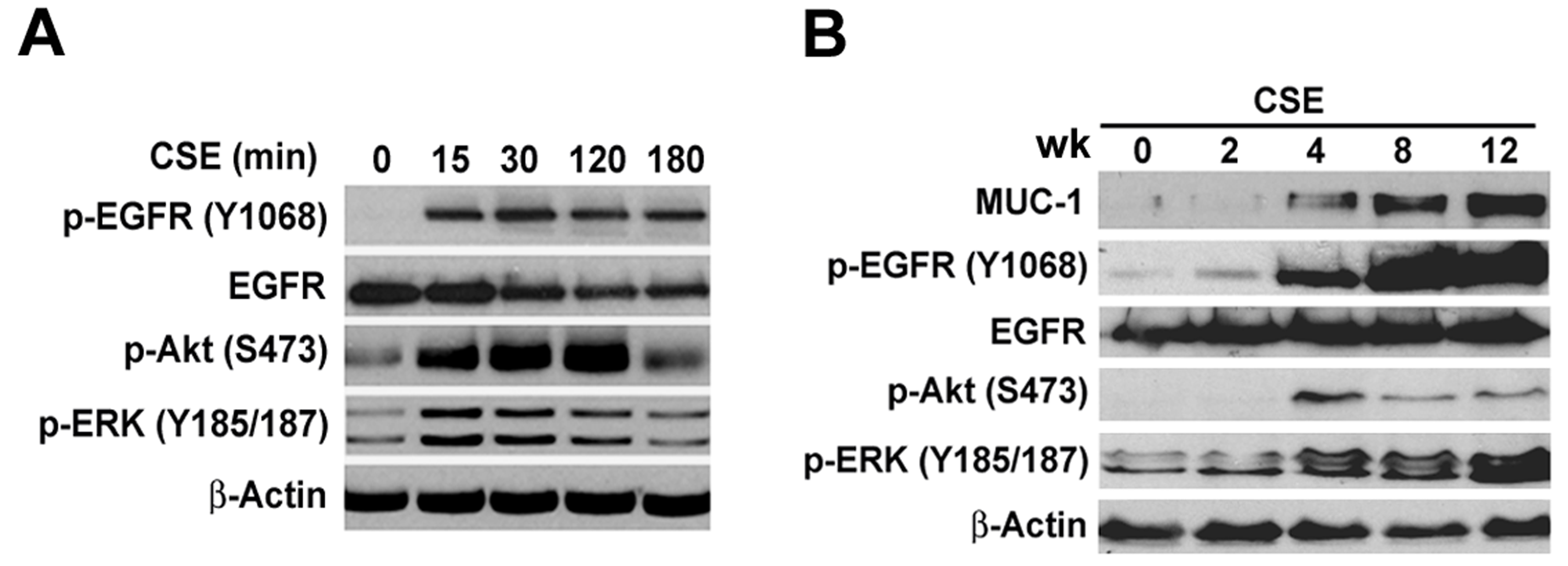

Supplement: Figure S2 — Induction of MUC1 expression and EGFR-, Akt- and ERK-activation in HBEC-2 cells by CSE exposure. A, HBEC-2 cells were treated with CSE (10 µg/ml TPM) for the indicated time periods. Activation of each protein was detected with antibodies against the phosphorylated form of the proteins. The phosphorylation sites of each protein were indicated. Total EGFR was also detected. β-Actin was detected as a loading control. B, HBEC-2 cells were treated with CSE (10 µg/ml TPM) for the indicated weeks. Western blot was same as in A. The expression of MUC1was also detected. β-Actin was detected as a loading control. (TIF) [file pone.0033846.s002.tif]

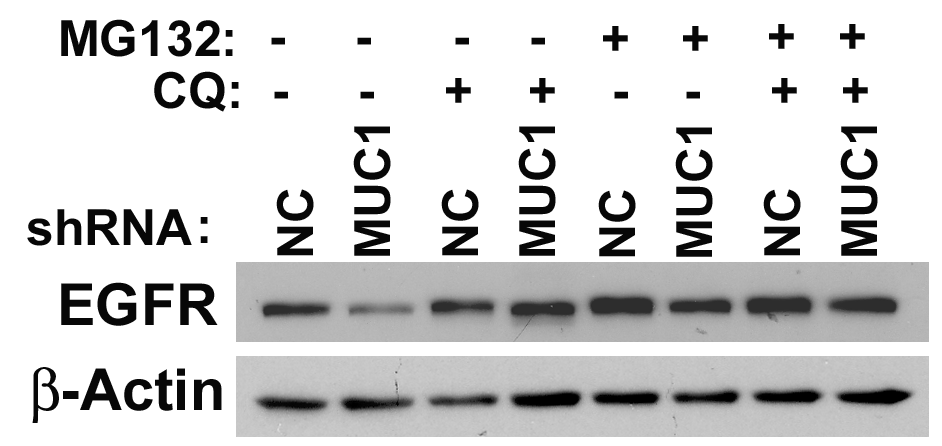

Supplement: Figure S3 — Blocking protein degradation with MG132 and chloroquine eliminates EGFR expression difference between the MUC1 knockdown and control cells. Beas-2B cells stably transfected with MUC1 shRNA or negative control (NC) shRNA were treated with lysosome inhibitor chloroquine (CQ, 20 µM), proteasome inhibitor MG132 (10 µM), or both for 15 h. EGFR protein was detected by Western blot. β-Actin was detected as an input control. (TIF) [file pone.0033846.s003.tif]
